# Combining high intensity ultrasound and experimental design to improve carotenoid extraction efficiency from Buriti (*Mauritia flexuosa)*

**DOI:** 10.1016/j.ultsonch.2022.106076

**Published:** 2022-06-21

**Authors:** Darlisson Slag Neri Silva, Matheus de Sousa Silva, Tiago Linus Silva Coelho, Clecio Dantas, Cícero Alves Lopes Júnior, Naise Mary Caldas, Edivan Carvalho Vieira

**Affiliations:** a Grupo de Instrumentação Analítica e Preparo de Amostra (GRIAPA), Department of Chemistry, Federal University of Piauí – UFPI, 64049-550 Teresina, Piauí, Brazil; b Laboratório de Química Computacional Inorgânica e Quimiometria – (LQCINMETRIA), State University of Maranhão - UEMA, 65604-380 Caxias, Maranhão, Brazil; c Grupo de Estudos em Bioanalítica – GEBIO, Department of Chemistry, Federal University of Piauí, 64049-550 Teresina, PI, Brazil; d Institute for Chemistry, TESLA – Analytical Chemistry, University of Graz, Universitätsplatz 1/I, 8010 Graz, Austria

**Keywords:** Buriti, Carotenoids, Chemometrics, Experimental designs, Ultrasound-assisted extraction, ANOVA, Analyses of variance, BBD, Box-Behnken design, CCD, Central composite design, CE, Conventional extraction, *F*_cal_, Calculated *F*, *F*_tab_, Tabulated *F*, LBF, Liquid biphasic flotation, rsd, relative standard deviation, RSM, Response surface methodology, SAM, Standard addition method, TCC, Total carotenoid content, UAE, Ultrasound-assisted extraction

## Abstract

Buriti (*Mauritia flexuosa* L.) is a significant source of carotenoids, but these compounds have been extracted using laborious and low-effective methods. The present work evaluated the high-intensity ultrasound combined with a chemometric approach to developing an optimal extraction method of carotenoids from buriti pulp. The multivariate optimization was carried out through two steps. First, a simplex-lattice mixture design was used to optimize the extractor solution finding higher extraction yield (903 ± 21 µg g^−1^) with the acetone:ethanol (75/25) mixture. After, sample mass (80 mg) and sonication time (30 min) were optimized applying central composite design (CCD) which provided a 14% improvement in the extraction method yield. So, the total carotenoid content (TCC) with optimal extraction conditions was 1026 ± 13 µg g^−1^ which is almost twice the yield of methods known in the literature for buriti. The RP-HPLC-DAD analysis revealed that the carotenoids are gently extracted and β-carotene is the major compound in the extracts. To confirm the accuracy, buriti samples spiked with β-carotene standard and the developed method showed recovery >84% and precision <6.5%. Furthermore, the optimized ultrasound-assisted extraction (UAE) method was applied to other samples (tomato, guava, carrot, mango, acerola, papaya, and pumpkin) and presented a yield to 5.5-fold higher when compared to the reported methods indicating high robustness. Based on results, the UAE method developed has demonstrated feasibility and reliability for the study of carotenoids in buriti pulp as well as in other plant matrices with high biological relevance.

## Highlights

• Buriti (*Mauritia flexuosa*) is a significant source of carotenoids.
• The simplex-lattice mixture design modified and CCD were used.
• The optimal condition for developed UAE was acetone/ethanol (3:1) and 80 mg of sample for 30 min.
• The yield (1026 ± 13 µg g^−1^) applying UAE was *ca.* 2-fold higher than known methods.
• UAE demonstrated feasibility and reliability for the study of carotenoids in different plant matrices.

## Graphical abstract

**Figure:**
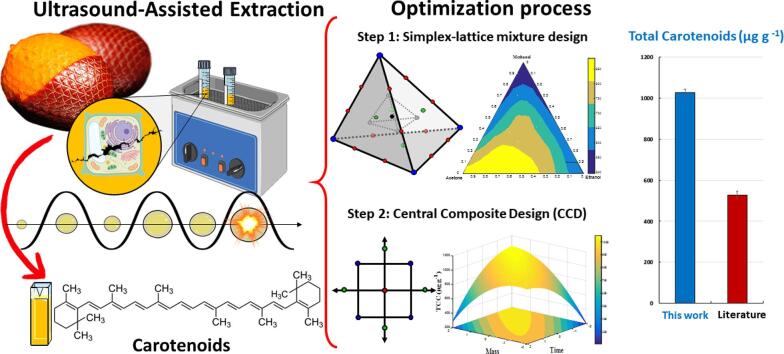

## 1 Introduction

Carotenoids are natural pigments synthesized by algae, plants, and photosynthetic bacteria [1]. These compounds have physiological functions in the human body and is also mainly intake through the diet. Buriti is the fruit and popular name of an abundant palm tree (*Mauritia flexuosa*) native to the Brazilian Cerrado biome and is also an excellent natural source of carotenoids with a much higher content *e.g.* carrots, mangoes, tomatoes, among others [2]. In addition to food, buriti has been used for cosmetic and pharmaceutical industry as a source of β-carotene, one of the most potent natural antioxidants [3]. Carotenoids have played an important role in different economic sectors producing USD 1.8 billion in 2018 and it has an estimated growth of 10% in the market for carotenoid-based products for 2022 [4]. So, research has been done focusing on new sources and more efficient methods extraction of carotenoids [5]. These compounds are susceptible to oxidation and isomerization during the extraction process from plant and animal matrices. This challenge can be overcome by combining a suitable extractor solution assisted by a soft energy (*e.g.* ultrasound) resulting in a gentle method for extraction these bioactive compounds [6], [7].

A wide variety of solvent combinations and extraction techniques have been used to carotenoid extraction: Soxhlet or maceration [8], microwave [9], the supercritical fluid extraction based on the use of supercritical CO_2_ [10], [11], liquid biphasic flotation (LBF) [12], [13] and enzyme-assisted extraction [14]. Some of these methods have some disadvantages like longer extraction times, high quantity of solvents used, higher associated cost, and heat degradation [7]. To overcome these difficulties, efforts are directed toward developing analytical methods that guarantee more ecologically friendly, sustainable, and viable techniques. Ultrasound-assisted extraction (UAE) is emerging and promising [15]. When compared to conventional extraction techniques, the UAE requires less equipment preparation time and allows the use of “recognized as safe” organic solvents, such as acetone and ethanol, without the need for prior adaptation of the equipment, making the extraction procedure more expensive and often makes the application of the methodology in industrial-scale unfeasible [16], [17]. The UAE is based on the action of low-frequency mechanical waves, which result in cavitation, a phenomenon responsible for the formation and collapse of cavitation microbubbles. During sonication, expand and compress their volume, causing pressure increase and heat release when imploding. This phenomenon promotes the breakdown of the cellular matrix and provides an increase in the release of the analyte [15], [18].

UAE can be performed using a bath or probe. The ultrasonic probe consists of a horn connected to a power transducer [19]. Although the ultrasonic probe is preferred over the bath system due to the higher ultrasonic intensity, the ultrasonic bath is a versatile alternative where the solid matrices are dispersed in the solvent in a stainless steel tank connected to the transducer [20], [21]. The ultrasound bath can reduce the operating temperature, allowing the extraction of thermolabile compounds besides that, it is more economical and easier to handle, but its low reproducibility restricts its use in the extraction process [15], [22]. In this context, chemometric approaches are powerful tools to promote the optimization and increase the reliability of extraction results in ultrasonic baths. The variables ultrasound power, sonication time, temperature, and ethanol percentage were optimized for the extraction of bioactive compounds from *Eucalyptus globulus* leaves by Box-Behnken design (BBD) [23]. A two-level factorial design showed that 49.2 % water content, ultrasound power of 72.4 W and sonication time of 9.7 min resulted in an optimal content of flavonoids extracted from *Paederia scandens* (Lour.)[24]. Box-Behnken is the most applied design for UAE optimization [25], [26]. However, the evaluation of the extraction mixture composition is crucial for the procedure's efficiency. Simplex-centroid design was applied on carotenoid extraction from the cashew apple, but the experimental design was not efficient in explaining the data set, which resulted in a lack of fit for mathematical modeling [27]. There are no reports of an optimized methodology for extracting total carotenoids from buriti in the literature. Through univariate studies using higher toxicity solvents such as hexane and petroleum ether and exhausting shaking, the values found for the total carotenoid content (TCC) varies from only from 446 µg g^−1^ to 529 µg g^−1^ [28], [29].

So, the objective of the present work was to develop a simple, efficient and reliable method for carotenoids extraction from buriti (*Mauritia flexuosa* L.) using ultrasound energy. A simplex-lattice mixture design modified was applied to minimize the effect of ultrasonic bath fluctuations on the results and to find the optimal extraction solution. To maximize yield, sample mass and sonication time were further optimized using a central composite design (CCD). The extracts were evaluated by RP-HPLC-DAD to investigate structural modification in carotenoids. The accuracy of the method was confirmed by the standard addition method using β-carotene while the robustness was evaluated by applying it in other well-studied plant matrices.

## 2 Material and methods

### 2.1 Samples

The samples of buriti were acquired from a local market at Caxias city (Maranhão, Brazil). The pulp samples (mesocarp) were obtained from ripe buriti fruit. Initially, the fruit skins were manually removed using a ceramic knife. Then, the pulp was collected with a simple scraping process from the peeled fruit and stored in a propylene tube at −4 °C.

### 2.2 Reagents and solutions

All reagents used in this work were of analytical grade or better. The materials were decontaminated in 10% (v/v) HNO_3_ acid bath for at least 24 h, then washed with deionized water (≥18.2 MΩ.cm) obtained in Milli-Q Direct purification system from Millipore® (Molsheim, France) and dried at room temperature. The solvents as acetone, methanol, acetonitrile were purchased form Sigma – Aldrich (Darmstadt, Germany) while ethanol was obtained from J.T.Baker® (Geel, Belgium). The concentration of solvents used in the runs was determined based on the lattice-simplex design applied as described in the section *2.4.1 Extractor solution*.

### 2.3 Ultrasound-Assisted extraction (UAE)

**Fig. 1:**
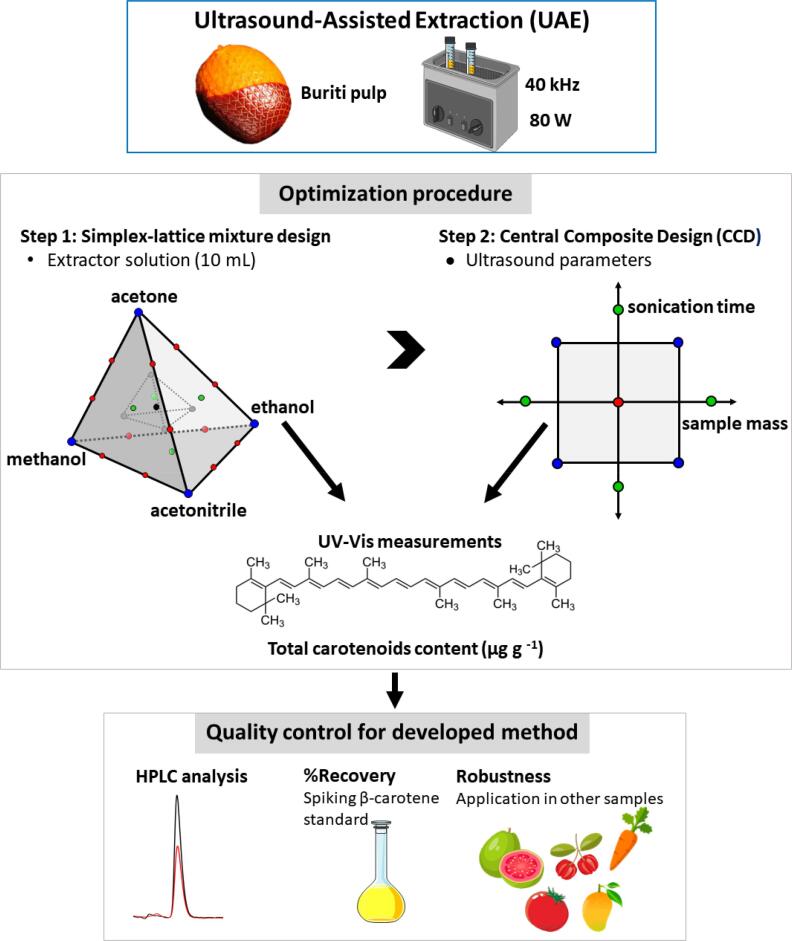
Workflow to develop the ultrasound-assisted carotenoid extraction method.

**Fig. 2:**
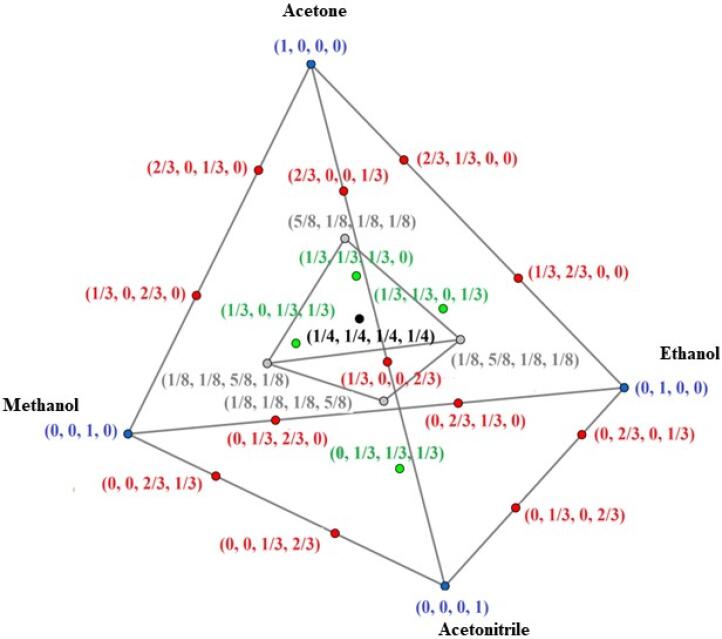
Simplex-lattice mixture design for extracting solution optimization. In parentheses, the solvent ratio for acetone, ethanol, methanol, and acetonitrile is shown.

Ultrasound-Assisted Extraction (UAE) was performed using an ultrasound cleaning bath (Sonitop 402-A, Soni-tech®, São Bernardo do Campo, SP, Brazil) operated at a frequency of 40 kHz and a power of 80 W with a usable volume of 1.9 L (internal dimensions 15 × 13 × 10 cm). Fig. 1 presents the whole methodology used to develop an optimized method for carotenoids extraction from buriti pulp. In the optimization step of the extractor solution, the sample mass was 100 mg weighed directly into a 15 mL polypropylene tube and sonicated for 15 min in the presence of 10 mL of solvent defined based on the simplex-lattice design applied (see Fig. 2). Then, the solution was centrifuged at 3500 rpm (1120 x*g*) for 10 min. The extraction temperature was kept in the range of 30–35 °C to avoid the degradation carotenoids and isomerization. The supernatant was collected in polypropylene tubes with wrapped aluminum foil and stored at –5 °C until further use. Total carotenoid content (TCC) was determined according to the method proposed by Tsiaka et al. [30], with some modifications.

### 2.4 Optimization procedure

In this study, a sequential optimization was performed to develop the ultrasound-assisted carotenoid extraction method as follows. Fig. 1 presents the method development workflow. First: use of the simplex-lattice mixture design to evaluate the most efficient composition of the extracting solution. Second: application of the CCD to verify the influence of the variables sample mass and sonication time and define the optimal extraction condition.

#### 2.4.1 Extractor solution

The acetone, ethanol, methanol, acetonitrile solvents were evaluated. These are the most commonly used solvents in reported work for the extraction of carotenoids from plant matrices in a univariate approach [6], [15]. So, a simplex-lattice mixture design of the type {4-component, 3-degree polynomial} with additional interior points as illustrated in Fig. 2 was applied evaluating the extraction yield for pure solvents and their mixtures.

As can be seen in Fig. 2, this system is symmetrically distributed in experimental points: four pure solvents, twelve binary mixtures, four ternary mixtures and five quaternary mixtures, resulting in 25 assays. The extraction using pure solvent is represented by blue points at the apex of the pyramid and also in terms of fractions, *e.g.* acetone as (1, 0, 0, 0), i.e. 100%. The extractions with binary solvents combinations were performed by the permutation of each participant in the percentage of 33.3% and 66.7%, in the pyramid of Fig. 2, these runs are arranged in space to occupy the lateral edges, highlighted in red, according to the (1/3, 2/3, 0, 0) distribution. For extractions using ternary mixture, the points are arranged on a pyramid face in green with 33.3% of each (1/3, 1/3, 1/3, 0). The interior gray points show combining the four solvents which one solvent is 62.5% in each experiment, and the other three are 12.5% ​​ (5/8, 1/8, 1/ 8, 1/8). Finally, the central black point corresponds to four solvents which it is represented in the center of the pyramid by (1/4, 1/4, 1/4, 1/4), *i.e*. 25%. All assays were performed randomly. The result for each assay is shown in Table 2.

The effect of simplex-lattice mixture design on the TCC was analyzed using linear, quadratic, special cubic and full cubic equations. To selecting the appropriate statistical model, analyses of variance (ANOVA) were employed. The best fit equation was obtained after removing the non-significant terms and the numerical optimization technique based on desirability function approach was used to optimize the mixture composition [31].

#### 2.4.2 Mass sample and sonication time

To maximize the efficiency of the method considering the highest TCC value extraction parameters such as sample mass and sonication time were also evaluated. So, a rotatable central composite design (CCD) was applied. The CCD was constituted of a full factorial 2^2^ design with the addition of four-star points between the axis plus repeat points at the centroid according to Table 1.

**Table 1:** Variables and value levels used in central composite design and total carotenoid content obtained.

Variables	Symbol	Variable Levels and Codes				
		-√2	−1	0	+1	+√2
Mass (mg)	(*x_1_*)	10	30	80	130	150
Time (min)	(*x_2_*)	14	20	30	40	44

The model quality and statistical significance of the coefficients were evaluated. The response surface methodology (RSM) was applied for modeling to maximize the extraction yield and to determine the optimal working condition [31].

### 2.5 Total carotenoid content (TCC)

The quantification of total carotenoid content extracted was performed based on the method developed by Tsiaka et al. [30] brief modifications, using a UV–Vis spectrophotometer (Genesys 10S UV–Vis, Thermo Scientific) and absorbance measurements at 455 nm. A stock solution of 500 µg mL^−1^ of β-carotene standard (Sigma - Aldrich, USA) in acetone was prepared by the dilutions of stock solution were made to produce the concentration ranging from 0.25 to 5 µg mL^−1^ used for the analytical curve. All experiments were performed in duplicate.

### 2.6 Evaluating extracts by RP-HPLC-DAD

The buriti pulp extracts obtained by applying the optimized extraction method was evaluated using an HPLC-DAD (Series 1260 infinity, Agilent Technologies, Waldbronm, Germany). A C18 HPLC column (Luna C18 (2) 5 µm, 250 × 4.6 mm) with 1 mL min^−1^ of mobile phase acetone:methanol (9:1, v/v) was used for the carotenoids analysis. The extracts were filtered with 0.45 µm hydrophobic PTFE (Polytetrafluoroethylene) syringe filters. The chromatographic profile of the extract was compared to the standard of β-carotene at a concentration of 100 mg L^−1^.

### 2.7 Validation of the optimal conditions

To confirm the accuracy of the developed method, an experiment with standard addition was performed which consists of spiking to sample with different analyte concentrations and evaluating the percentage that was recovered at the end of the extraction process. Then, buriti samples were spiked with three β-carotene concentrations such as 50, 100 and 150 µg g^−1^ and submitted to the optimal extraction condition. The precision was determined in terms of repeatability considering the relative standard deviation (rsd) while the accuracy was determined as the ratio between the average concentration for the sample without spiking and the expected concentration after the spiking and expressed as a percentage of analyte recovered after application of the UAE method developed.

Also, robustness was evaluated. Then, the UAE method developed was applied to other well-studied plant samples. Frozen samples of tomato, guava, carrot, mango, acerola, papaya and pumpkin were thawed in a refrigerator at 4 °C. After the samples thawed, the outermost layer such as husk and seeds were removed. Homogenization was carried out separately in a food microprocessor (Philips Walita, RI7630) until the sample presented the consistency of a fine homogeneous paste. The carotenoid extraction was carried out applying the optimal conditions of the method as follows: 80 mg of sample in the presence of 10 mL of acetone:ethanol (75/25) extractor solution under 30 min of sonication in an ultrasonic bath. After that, the solution was centrifuged for 10 min at 3500 rpm (1120 x*g*). The TCC was quantified in the extracts as described in *item 2.4*.

### 2.8 Statistical analysis

The statistical analysis of data obtained and mathematical calculations of models generated were performed by employing the MATLAB software (version R2015a). All analyses were done in duplicate and the replicates provided the degrees of freedom to calculate the pure error and the lack of fit from the models. All experiments were performed randomly. The comparisons were all accomplished with a confidence level of 95%. Tukey test was performed to evaluate the significance of the differences between mean values for each assay.

## 3 Results and discussion

### 3.1 Multivariate sequential optimization

#### 3.1.1 Extractor solution composition

Although most studies on carotenoid UAE have focused on optimizing variables (*e.g.* mass, time, potency, among others), the extractor solution composition is crucial for the success of the extraction method [6], [17], [23], [32]. So, in this work was proposed a simplex-lattice design to evaluate the solvents acetone, ethanol, methanol and acetonitrile as well as their combinations in binary, ternary and quaternary mixtures resulting in 25 experiments as shown in Fig. 2. The solvents chosen are the most used for the extraction of carotenoids in plant matrices and compatible with ultrasound energy [15]. Table 2 shows the TCC values found for the assays carried out based on simplex-lattice design.

**Table 2:** Total carotenoid content (µg g^−1^) in the assays carried out based on simplex-lattice design.

Assay	Acetone (a)	Ethanol (b)	Methanol (c)	Acetonitrile (d)	TCC (µg g^−1^)	
					R1	R2
E1	1				830	837
E2		1			588	593
E3			1		430	445
E4				1	414	419
E5	1/3	2/3			770	786
E6	1/3		2/3		529	518
E7	1/3			2/3	478	470
E8		1/3	2/3		583	560
E9		1/3		2/3	531	519
E10			1/3	2/3	563	539
E11	2/3	1/3			888	918
E12	2/3		1/3		619	638
E13	2/3			1/3	623	621
E14		2/3	1/3		597	580
E15		2/3		1/3	552	541
E16			2/3	1/3	569	580
E17	1/3	1/3	1/3		723	724
E18	1/3	1/3		1/3	787	811
E19	1/3		1/3	1/3	695	672
E20		1/3	1/3	1/3	541	639
E21	5/8	1/8	1/8	1/8	801	809
E22	1/8	5/8	1/8	1/8	684	675
E23	1/8	1/8	5/8	1/8	603	599
E24	1/8	1/8	1/8	5/8	605	590
E25	1/4	1/4	1/4	1/4	753	782

As can be seen in Table 2, TCC values ranged from 414 to 918 µg g^−1^. Considering the pure solvents, acetone showed the highest TCC values (E1 – 833 ± 5 µg g^−1^) and was 2-fold higher than acetonitrile (E4 – 416 ± 3 µg g^−1^). This result is related to the solubility of carotenoids in solvents which the order is as follows: acetone (200 mg L^−1^) > ethanol (30 mg L^−1^) > acetonitrile and methanol (10 mg L^−1^) [33]. The extraction of total carotenoids from raw carrot, sweet potato and chicken nuggets using acetone also showed a higher TCC value than acetonitrile and methanol [34]. Pure acetone was also more efficient to extract carotenoids from paprika by shaking [10]. However, some studies demonstrate that the extraction of bioactive compounds is more efficient with the combination of solvents [23], [32]. Observing Table 2, the highest TCC value found was with the binary mixture acetone:ethanol (E11 – 918 µg g^−1^). Solvent polarity has a significant effect on carotenoid extraction. Buriti has a high predominance of nonpolar carotenoids [28], [35]. On the empirical quantitative scale of E_T_(30) polarity, the values for acetone, ethanol, methanol and acetonitrile are 42.2, 51.9, 55.4, and 45.6 kcal.mol^−1^, respectively [36]. Therefore, the estimated polarity for the acetone:ethanol mixture is intermediate. This behavior can have contributed to the high TCC value found in the E11 assay. The ternary mixture of acetone, ethanol and hexane defined by applying a centroid-simplex design was the most efficient for lycopene extraction from samples of raw tomato, sauce and paste [37]. For pigments extraction from *Coffea arabica* L. leaves, the combination of ethanol, hexane and dichloromethane was the best extractor solution composition [32]. These findings demonstrate that the extractor solution composition depends on the target carotenoids and the plant matrix.

In addition, the penetration capacity of the extractor solvent combined with its interaction with ultrasonic waves affects the carotenoid extraction. Acetone and ethanol present high permeation in plant tissues and then extract more bioactive compounds due to the increase in the solvent/solute ratio [38]. In a study on UAE of carotenoids from *Heterochlorella luteoviridis* was found a higher yield using 75% of ethanol concentration under 80% of ultrasound intensity [[12], [39]]. In fact, the effect of ultrasonic waves causes cavitation in the extracting solvent releasing high energy (5000 K, 1000 atm) with rupture of its bubbles which contributes to cell lysis and consequently the solubility ratio increasing the extractability of the target bioactive compounds [15], [25], [40].

The TCC values found (Table 2) for each assay of the experimental mixture design were considered the analytical response for statistical modeling. The performance of the linear, quadratic, special cubic and full cubic model was assessed using analysis of variance (ANOVA). All models captured a significant variance at 95% confidence level (*F*_cal_ > *F*_tab_). However, the special and full cubic were the only ones that did not show lack of fit (*F*_cal_ < *F*_tab_). The special cubic model was selected because the small improvement in predictive capability from full cubic is not a rationale for the use of a more complex model. Table 3 presents ANOVA for the special cubic model proposed considering the significant terms. As can be seen, the model is well-fitted, reliable (*F*_cal_ (146.68) ≫ *F*_tab_ (2.00)) and high predictive describing *ca.* 98.15% of data variance.

**Table 3:** Analysis of variance (ANOVA) for special cubic model.

Source of variation	Sum of squares	Degrees of freedom	Mean square	*F*_cal_	*p*-value	*F*_tab_*
Model	765264	13	58866	146.68	< 0.00001	2.00
Residual	14448	36	401			
Lack of fit	6593	11	599	1.91	0.0879	2.20
Pure error	7855	25	314			
Total adjustment	779712	49	15913			
R^2**^	0.9815					
R^2^_ajust***_	0.9748					

*p = 0.05.

^**^ coefficient of determination.

^***^ adjusted coefficient of determination.

The special cubic model proposed is represented by the polynomial described in Eq. (1):

(1) TCC=839.56a+570.75b+447.03c+417.58d+603.72ab--300.35ac--360.51ad+308.97bc+176.81bd+583.71cd+956.81abc+3845.62abd+3419.77acd

Eq. (1) was considered only the significant terms. All linear coefficients were significant and positive which primary solvent effect of acetone (*a*) was the most important. Most effects of the mixtures are synergistic, except for the mixtures acetone:methanol (*ac*) and acetone:acetonitrile (*ad*) are antagonistic. Generally, the carotenoid extraction from plant matrices has a higher yield using a combination of solvents [6], [9], [21].

**Fig. 3:**
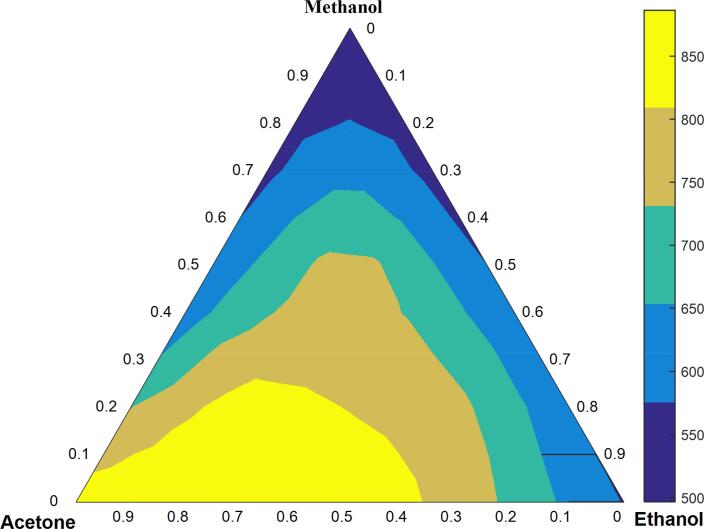
Contour surface for the special cubic model which the concentration of TCC is predicted as a function of the ratio of acetone, ethanol, and methanol solvents.

Fig. 3 shows the surface contour plotted applying Eq. (1) which illustrates the effect of extractor solution composition on total carotenoid concentration. As can be observed, the highest TCC values (>800 µg g^−1^) are in the vicinity of an acetone:ethanol binary mixture that is richer in acetone. The numerical analysis indicated the best solvent mixture for the extraction of carotenoids from buriti pulp would be 75% of acetone and 25% ethanol, with an optimal TCC of 886 µg g^−1^ (desirability of 0.936).

Buriti pulp is rich in non-polar and polar carotenoids [28], [35] acetone and ethanol mixture solution is more suitable for the study of bioactive compounds in this plant matrix. Add to that, the fact that these solvents also have a high capacity for penetrating cells and interacting with ultrasonic waves, favoring cavitation [15]. Up to this point, the mixture design methodology provided the composition of the optimal extractor solution.

#### 3.1.2 Optimizing of sample mass and sonication time by central composite design

**Fig. 4:**
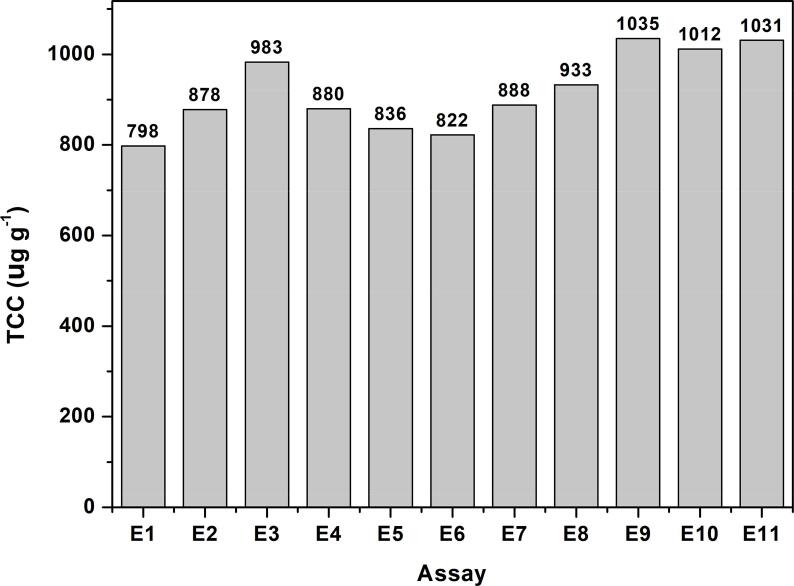
Total carotenoid content (µg g^−1^) found for the 11 trials proposed by the central composite design.

As can be seen in Fig. 1, in this work a sequential multivariate optimization was applied to develop a UAE method of carotenoids from buriti pulp. The acetone:ethanol mixture (75/25) was defined as the best extractor solution based on the applied simplex-lattice mixture design. However, it is also known that sample mass and sonication time have a significant effect on the UAE of carotenoids [41], [42]. Then, these parameters were optimized using a central composite design (CCD) as described in Table 1. The Fig. 4 shows the TCC values found for the 11 trials proposed by the experimental design.

Observing Fig. 4, the TCC values found ranged from 798 to 1035 µg g^−1^. Considering the maximum TCC values, the application of CCD resulted in *ca.* 17% increase in extraction yield compared to simplex-lattice mixture design, confirming that sample mass and sonication time are significant parameters. The results shown in Fig. 4 were fitted to a quadratic regression model. The polynomial equation is represented by Eq. (2) which only significant coefficients were used as follows:

(2) $$\mathrm{TCC}\;=\;1026.00\;+\;21.00x_{1}-94.74x_{1}^{2}-53.94x_{2}^{2}-45.70x_{1}x_{2}$$

The quality of the model was evaluated using ANOVA (Table 4). The established model was found to be significant (*F*_cal_ = 13.81 and *p* < 0.05), useful to predict the total carotenoid content showing no significant lack of fit (*F*_cal_ = 11.12 and *p* > 0.05) and describing 90% of the total variance.

**Table 4:** Analysis of variance (ANOVA) for central composite design.

Source of Variation	Sum of squares	Degrees of freedom	Mean square	*F*_cal_	*p*-value	*F*_tab_*
Model	66769	4	16692.26	13.81	0.003	4.53
Residual	7253	6	1208.81			
Lack of fit	6944	4	1735.18	11.12	0.0842	19.25
Pure error	312	2	156.07			
Total adjustment	74022	10				
R^2^	0.90					
R^2^_ajust_	0.84					

* *p* = 0.05.

**Fig. 5:**
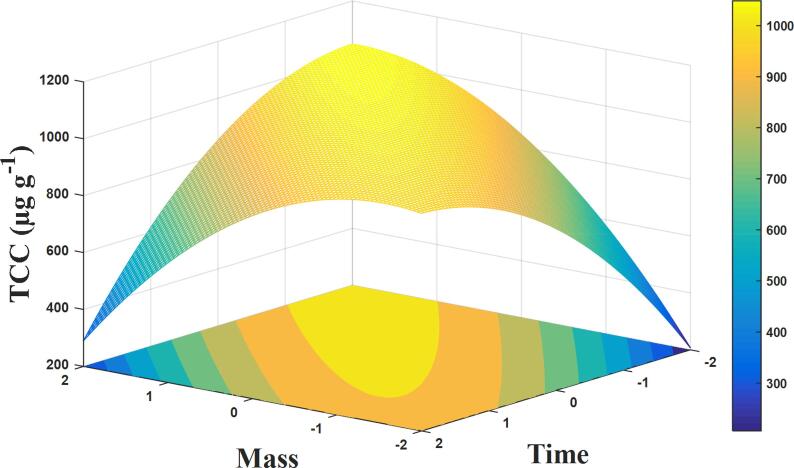
3D response surface of total carotenoid content from the CCD using proposed quadratic model.

The response surface plotted for the effect of mass (*x_1_*) and sonication time (*x_2_*) on the extraction yield for UAE obtained by fitting the quadratic model expressed by Eq. (2) is show in Fig. 5. As can be seen, the highest total carotenoid content is in the region of central point. The numerical analysis indicated the best extraction of carotenoids from central composite design would be 80 mg of sample mass and 30 min of extraction time, with an optimal TCC of 1026 µg g^−1^ (desirability of 0.961).

Regarding the sample mass, some works have been developed focusing on the optimization of this parameter. Coelho et al. [31] optimized the carotenoid extraction in cashew apple, evaluating the range from 59 to 201 mg. The optimal condition was 135 mg and 153 mg for conventional extraction and UAE, respectively. These values are up to 91% higher compared to our study.

Short sonication time has a low effect on the extraction of carotenoids from buriti pulp. TCC values increase from 20 to 30 min, i.e. between level −1 and 0. This behavior is similar to the extraction of bioactive compounds from the olive leaf using ultrasound energy [43]. So, the mechanism of the UAE process of buriti carotenoids may occur in two steps. Initially, the effect of cavitation promotes cell lysis and dissolution of carotenoids which occurs fast as “washing”. The second step is slower, which consists of the mass transfer of the solute from plant tissues to the solvent by diffusion or osmotic process [43], [44]. Longer sonication time reveals a tendency of reduction in TCC values. This fact can be attributed to the degradation of carotenoids in buriti extracts due to high exposure to ultrasound radiation [41], [45]. Also, a long sonication time is unattractive, reducing the analytical frequency and increasing the cost of the method. Observing some reported studies, the sonication time may be related to the plant matrix. For example, the recovery of β-carotene from a pumpkin by UAE using mixed eutectic solvents was carried out in 10 min [17]. The Box–Behnken design (BBD) was used to optimize some independent factors such as ultrasonic intensity (50–90 W/m^2^), sonication time (5–20 min), and solid-solvent ratio (1.5–30%, w/v) in the extracted lutein content from marigold petals which sonication for 12 min was adequate to achieve the highest yields [7]. The UAE of carotenoids from orange peel by vegetable oils required 35 min of sonication to provide the highest yields indicated by CCD [46]. On the other hand, sonication for 5 h was required to achieve high lutein content (3.16 ± 0.03 mg g^−1^) in extracts from *Chlorella vulgaris* [47].

In comparison, the TCC value found using optimized UAE here is 2.0-fold higher than the known values reported for buriti pulp [35], [48]. For exemple, Lima et al*.* [48] found a TCC in buriti from Goiás state of 446 µg g^−1^ in the maceration procedure from the fresh sample using acetone and then partitioned with petroleum ether, being concentrated by evaporating the solvent under N_2_ stream. De Rosso & Mercadante [35] used only acetone to extract, after the exhaustive extraction, the solution was transferred to petroleum ether/diethyl ether and saponified with KOH this procedure allows obtaining a TCC of 514 µg g^−1^ for the buriti from the Amazon region.

So, the application of sequential multivariate optimization was successful combining simplex-lattice mixing design to select the extracting solvent and central compound design to optimize sample mass and sonication time. The developed method is fast, simple, gentle and efficient for carotenoids extraction from buriti pulp.

### 3.2 Quality control

#### 3.2.1 RP-HPLC-DAD analysis

**Fig. 6:**
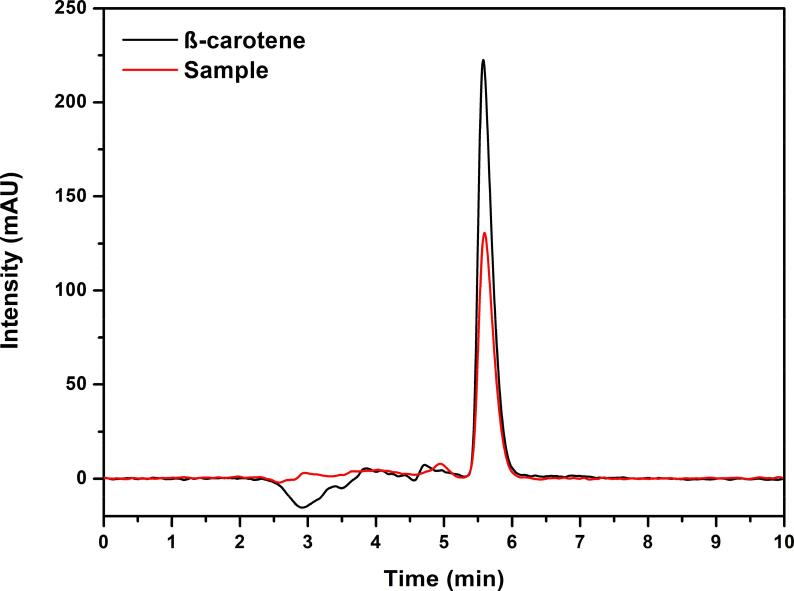
Chromatogram for the β-carotene standard and the buriti extract provided using UAE optimized method.

Although the ultrasound bath produces a low intensity cavitation when compared to the ultrasound probe, the degradation of carotenoids can happen during the extraction process [19]. The buriti extracts obtained with the optimized UAE method as follows: 80 mg of sample in the presence of 10 mL of acetone:ethanol (75/25) sonicated for 30 min were evaluated by RP-HPLC-DAD. Fig. 6 shows the chromatogram of the buriti extract and the β-carotene standard. The total time for the run was 10 min and 5.6 min for the retention time of the highest intensity compounds signal.

As can be seen in Fig. 6, the buriti extract obtained with optimal extraction conditions showed that there was no degradation of the compounds during the UAE. The developed method allows gentle extraction of carotenoids of the buriti. This behavior was also observed by Goula et al. [45], who, through the analysis of chromatograms of carotenoids extracted from pomegranate residues using vegetable oils, showed that there was no degradation by the ultrasonic cavitation process. In contrast, the yield of β-carotene extraction from citrus peel using an ultrasound probe (frequency 21–25 kHz, 10 min) decreased with increasing time, and the degradation products were identified by HPLC-DAD analysis [49].

The investigation of the extracts using RP-HPLC-DAD also revealed that β-carotene is the major carotenoid present in the extracts. This result is expected comparing with the chromatographic profile of the buriti extract reported in previous studies [35]. So, the β-carotene standard was selected to confirm the accuracy of the method by the standard addition assay.

#### 3.2.2 Standard addition method

To ensure that the developed ultrasound-assisted extraction method presented reliable results, the accuracy was evaluated by applying the standard addition method (SAM). In this approach, a known quantity of analyte (β-carotene) was added to the sample, and at the end of the analytical procedure, the remaining content in the analytical portion was evaluated [34]. The recovery percent at spiked 50, 100 and 150 µg g^−1^ concentrations were 84.4%, 84.0% and 98.0%, respectively. Therefore, the recovery percent at all levels was greater than 80%, indicating adequate accuracy for the developed method. Furthermore, these results corroborate those of the RP-HPLC-DAD analysis, confirming minimal oxidation and isomerization of the bioactive compounds in the buriti extracts provided with the optimized method.

The precision of the proposed SAM was evaluated considering the relative standard deviation (rsd). The rsd values were in the range of 1.6 to 6.5%. These findings show that the developed method is accurate and precise.

#### 3.2.3 Evaluating other plant samples

The efficiency of the method developed was evaluated by studying other plant matrices. So, seven representative fresh samples as follows: tomato, guava, carrot, mango, acerola, papaya, and pumpkin, were used to verify the applicability of the optimized UAE method. Table 5 shows the total carotenoid content found in the different plant matrices using the developed method and the results reported from previous works [50], [51], [52], [53], [54], [55], [56]. In comparison, the TCC values found in the plant matrices studied here were higher than the values reported using other methods ranging from 1.2 to 5.5-fold, except for pumpkin. However, de Carvalho et al. [55] used 15 g of pumpkin and 3 × 15 mL of acetone under agitation and then the extractor solvent was removed using a separatory funnel with 40 mL of petroleum ether. This extraction procedure is much more laborious and expensive compared to the method developed in this work. Indeed, the proposed UAE of carotenoids demonstrated robustness and more efficient than previous methods.

**Table 5:** Total carotenoid content present in representative samples (fresh weight – FW).

Sample	Total Carotenoid Content (µg g^−1^)		Extraction Method	Ref
	Our study	Literature		
Tomato	197 ± 16	144 ± 1	Mechanical extraction, 20 mg freeze-dried sample + 250 µL of methanol + 500 µL of trichloromethane and 250 µL of deionized water	[51]
Red Guava	131 ± 12	24 ± 1	Exhaustive mechanical extraction with 5 g of sample plus acetone and saponified overnight with 10% (w/v) KOH in methanol overnight at room temperature	[56]
Carrot	221 ± 15	194 ± 11	Ultrasound extraction, 2 g of sample, 10 mL of hexane:acetone:ethanol (2:1:1, v/v) + 0.1% BHT + 0.02 g of MgCO_3_. Sonicated for 5 min, centrifuged for 5 min at 5000*g*. Hexane phase was re-extracted 3 times with 10 mL of hexane with 0.1% BHT	[50]
Mango	30 ± 2	19 ± 1	Mechanical extraction, 10 g of sample + acetone:petroleum ether (4:1, v/v) + 0.1% BHT. Centrifuged for 20 min at 10,000*g*. The supernatant was purified into a separatory funnel and 75 mL of 20% (w/v) NaCl was added.	[53]
Acerola	118 ± 9	25 ± 3	Mechanical extraction, 1 g of sample was triturated with 60 mL of cold acetone, vacuum filtered and transferred to a cold petroleum ether (50 mL). Rotary evaporated and the carotenoids were dissolved in 25 mL petroleum ether	[52]
Papaya	26 ± 2	10.1 ± 0.3	Mechanical extraction, 15 g + acetone, after was transferred into light petroleum:diethyl ether (2:1), saponified overnight with 10% methanolic KOH, washed and rotary evaporated	[54]
Pumpkin	132 ± 10	234 ± 1	Mechanical extraction, 15 g of sample + 3 g of celite, successive extractions with 25 mL of acetone, extract was transferred to a separatory funnel containing 400 mL of petroleum ether, acetone was removed with ultrapure water and the extract was dehumidifier with anhydrous sodium sulfate.	[55]

Comparing the total carotenoid content found in the different plant samples studied here, buriti pulp presented a value 5.3, 7.9, 4.7, 34, 8.8, 39, 7.8-fold higher than tomato, guava, carrot, mango, acerola, papaya, and pumpkin, respectively. Buriti is the richest plant matrix in carotenoids which β-carotene is the most abundant species.

## 4 Conclusions

The proposed objectives for the work were satisfactorily achieved, resulting in the development of a reliable and efficient method for extracting carotenoids from buriti pulp. Sequential multivariate optimization was a crucial approach to maximize the efficiency of the developed method. For instance, the simplex-lattice mixture design was decisive to overcome the fluctuations in the results due to the ultrasonic bath and to achieve a regression model fitting indicating acetone:ethanol (75/25) as the optimal extracting solution. The UAE method developed showed a yield (1026 ± 13 µg g^−1^) *ca.* 2-fold higher than the methods known in the literature for buriti. The RP-HPLC-DAD analysis revealed that the carotenoids are gently extracted and β-carotene is the major compound in the extracts. To confirm the accuracy, buriti samples spiked with β-carotene standard and the developed method showed recovery > 84% and precision < 6.5%. Furthermore, the optimized UAE method was applied to other plant samples and presented a yield to 5.5-fold higher when compared to the reported methods indicating high robustness. Therefore, the UAE method developed has demonstrated feasibility and reliability for the study of carotenoids in buriti pulp as well as in other plant matrices with high biological relevance.

For future studies, some ultrasound parameters such as power and frequency can be evaluated by applying a multivariate optimization. In fact, this can increase the efficiency of the carotenoid extraction method, but a more sophisticated ultrasonic bath is required. Also, it is important to investigate using LC-MS to characterize better the carotenoid compounds present in the buriti extracts provided from the proposed UAE.

### CRediT authorship contribution statement

**Darlisson Slag Neri Silva:** Formal analysis, Conceptualization, Visualization, Writing – original draft. **Matheus de Sousa Silva:** Methodology, Formal analysis. **Tiago Linus Silva Coelho:** Formal analysis. **Clecio Dantas:** Methodology, Data curation, Conceptualization, Visualization. **Cícero Alves Lopes Júnior:** Funding acquisition, Conceptualization, Resources, Visualization, Writing – review & editing. **Naise Mary Caldas:** Funding acquisition, Conceptualization, Project administration, Supervision, Visualization. **Edivan Carvalho Vieira:** Funding acquisition, Project administration, Resources, Supervision, Visualization, Writing – review & editing.

## Declaration of Competing Interest

The authors declare that they have no known competing financial interests or personal relationships that could have appeared to influence the work reported in this paper.
